# Community-engaged research in HIV implementation science: A cross-sectional assessment of meaningful engagement among community and academic recipients of 2021 and 2022 ‘ending the HIV epidemic’ supplement awards

**DOI:** 10.21203/rs.3.rs-8554220/v1

**Published:** 2026-01-22

**Authors:** Reva Datar, Wilson Gomez, Sheree Schwartz, David A Katz, April Petit, Audrey Harkness, Jonathan Ross, Pedro Alonso Serrano, Stefan Baral, Laura Beres

**Affiliations:** Johns Hopkins Bloomberg School of Public Health; Johns Hopkins Bloomberg School of Public Health; Johns Hopkins Bloomberg School of Public Health; University of Washington; Vanderbilt University; University of Miami; Albert Einstein College of Medicine; Chicago Queer Latine Collaborative NFP; Johns Hopkins Bloomberg School of Public Health; Johns Hopkins Bloomberg School of Public Health

**Keywords:** community-engaged research, HIV, implementation science

## Abstract

**Background:**

Meaningful community engagement is fundamental to sustained and effective implementation of HIV interventions, yet partnership engagement quality is rarely measured. We assessed meaningful engagement among community and academic partnerships from 2021-2022 United States Ending the HIV Epidemic (EHE) Implementation Science (IS) Awards.

**Methods:**

Awardees were invited to complete a cross-sectional, online survey. We applied adapted, validated community engagement importance and quality measures across six ‘engagement principles’ (EPs) and 12 attributes of ‘trust’ using 5-point Likert scales (1=‘poor’ to 5=‘excellent’). Level of community partner engagement was assessed along a continuum. Differences in EP scores between academic partners and community partners were assessed using Mann-Whitney U Test. Bivariate associations between engagement level, EPs, and trust used Spearman’s rank correlations. Open-ended responses were thematically analyzed.

**Results:**

From July-December 2023, we received 53 (65%) surveys from academic partners and 29 (35%) from community partners, including 1:1 paired responses for 18/57 (32%) projects. Community partners and academic partners reported high median quality engagement summary scores (3.6 vs 4.1, p=0.04) and trust scores (>67% and >69% reporting “very good” or “excellent”). All trust attributes were highly valued, with >85% of participants rating each “important” or “very important.” Academic partners reported lower median summary scores than community partners for quality and frequency of EPs [3.6 vs 4.1, p=0.04; 3.8 vs 4.3, p=0.02]. ‘Fostering co-learning’ was the only individual EP scored significantly lower by academic than community partners (quality [−2.6, p=0.01], frequency [−2.6, p=0.01]). For trust, academic partners reported the lowest quality scores for dependability, while community partners reported the lowest for mutual benefit. Among academic partners, level of community engagement was positively correlated with quality across all six EPs (p<0.05). While among community partners, quality of all trust attributes were positively correlated with EP quality (p<0.5). In open-ended responses, structural barriers to engagement and concern about community partner burden were prominent themes.

**Conclusions:**

EHE awards largely supported trusting and meaningful partnerships. Differences between academic and community responses indicate a need to develop shared understanding of engagement quality. Strategies to strengthen trust, support greater engagement, and enhance the engagement quality will optimize partnerships and their potential positive impact on implementation and effectiveness outcomes.

## Background

Community engagement in HIV research has supported identification of key health priorities, championed inclusive and impactful research studies, and implemented interventions to improve the lives of those living with or impacted by HIV [1]. Over the past four decades, HIV science has made significant progress in the development and availability of effective treatment and prevention interventions such as novel, long-acting injectable antiretroviral therapy (ART) and pre-exposure prophylaxis (PrEP) [2]. However, persistent challenges implementing these interventions in diverse, real-world settings have contributed to entrenched health inequities and a continuing HIV epidemic in the United States (US). Quality community engagement is widely recognized as a critical factor for successful implementation research but is infrequently measured among community and academic partners [3]. Optimizing the frequency and quality of researcher and community partner research collaborations may improve implementation efforts, ameliorating health inequities and outcomes.

To advance the impact of HIV treatment and prevention tools, technologies, and programs, the national Ending the HIV Epidemic (EHE) initiative was launched in 2019 with a goal of reducing new HIV diagnoses in the US by 90% by 2030 [4, 5]. As part of this initiative, the National Institutes of Health (NIH) supported 1-2-year awards funding HIV implementation research via community-academic collaborations to generate evidence for improved HIV treatment and prevention efforts [6, 7]. Academic-based recipients of these research awards were required to collaborate with a community-based partner. To date, nearly 250 implementation research projects have been funded under the NIH EHE Implementation Science (IS) initiative [8]. To provide support and expertise in IS to funded community-academic partnerships, NIH also funded EHE Regional Consultation Hubs and the Implementation Science Coordination Initiative (ISCI; www.HIVimpsci.org).

Community-engaged research (CEnR), defined as “the process of working collaboratively with and through groups of people affiliated by geographic proximity, special interests, or similar situations to address issues affecting the well-being of those people,” [9] is growing in implementation science. However extant literature is limited in its exploration of how perceptions of CEnR among partner entities (i.e., academic partners and community partners) engaged in IS projects may align or differ and how concurrence contributes to research processes and outcomes. Recent studies of NIH-funded HIV IS projects identified improved community engagement as a critical next step to the scale-up, dissemination, and delivery of HIV interventions [1, 8, 10, 11]. Our previous research on CEnR among community partners and academic partners engaged in EHE awards found that while the EHE awards largely supported active community engagement in research activities, frequent challenges included mis-matched expectations for the partnership, funding and reimbursement mechanisms appropriate for academic institutions but ill-fit for community partners, and limited community partner participation in foundational activities including grant writing [6, 12]. Evaluations of CEnR models have highlighted the role of substantive or meaningful engagement and trust in the success of community-academic partnerships [13-15]; however, many of these studies were outside of IS [16-19], used primarily qualitative methods [6, 15, 20, 21], or omitted community partner perspectives from quantitative measurement [17, 19, 22]

The EHE NIH IS supplement awards offer a unique opportunity to examine perceptions of CEnR in the context of HIV IS among both community and academic partners. To inform strategies for improved community-academic IS partnerships, our study objective was to quantitatively assess: 1) the quality and frequency of meaningful partner engagement, and 2) the quality and importance of perceived trust among community and academic partner recipients of EHE supplement awards.

## Methods

### Sample and setting

This study used a cross-sectional survey to examine CEnR practices in HIV-related implementation science. Eligible survey respondents were adult (≥ 18 years) members of an academic or community partner organization awarded an EHE NIH IS Award (hereafter referred to as EHE Awards) in 2021 or 2022 who self-identified as having significant project involvement, defined as “frequent and in-depth involvement” in the EHE project.

We invited prior recipients of EHE Awards to join our study’s Community Engagement Advisory Committee. Nine academic investigators joined the committee prior to study onset with an additional nine community partner representatives joining after data collection was completed. Advisory Committee members provided guidance on study design, survey development, pilot testing, and results interpretation.

### Measures

We developed a 65-question self-report online survey consisting of engagement measures adapted from prior studies (see Supplemental Material for complete survey, S1) [23-25]. In a previous study using a subset of these 65 questions, we assessed anticipated and experienced levels of community engagement and community engagement throughout the research process to characterize community-academic partnerships supported through EHE Awards [12]. Here, we examine the measures pertaining to perceptions of meaningful and trustworthy engagement in the required EHE partnerships:

#### Demographics

Participants self-reported their age in years, gender, highest educational attainment, race, and ethnicity.

##### Meaningful Engagement:

Adapting Goodman et. al.’s (2017) Quantitative Community Engagement Measure [12, 24], we assessed participants’ level of engagement in their EHE project across six ‘Engagement Principles’ (EPs). Each EP was measured using four to six specific survey items. Each item was assessed for perceived frequency (i.e., how often an EP-specific action occurred within the partnership) and perceived quality (i.e., the participant-assessed quality of engagement for each EP-specific action). EPs included: (1) Disseminate findings and knowledge gained to all partners; (2) Foster co-learning, capacity building, and co-benefit for all partners; (3) Integrate and achieve a balance of all partners; (4) Seek and use the input of community partners; (5) Facilitate collaborative, equitable partnerships; and (6) Involve all partners in the dissemination process. Response options for frequency items of each EP were: 1 = Never 2 = Rarely, 3 = Sometimes, 4 = Most of the Time, 5 = Always and for quality were: 1 = Poor, 2 = Fair, 3 = Good, 4 = Very Good, 5 = Excellent. Participants could respond with “Not Applicable” if they chose. For each participant, we summarized responses into: 1) EP-specific scores on each scale (quality and frequency for each EP) by averaging the scores of the relevant items used to measure each EP and 2) Total quality and frequency scores by averaging the EP-specific scores on each scale. These summary scores were used in subsequent analyses. An additional qualitative, free text response question asked, “Are there any other components of meaningful engagement that you think should be included or addressed in this survey? If so, please describe in the box below:”

##### Partnership Trust:

Adapting the Center for Disease Control and Prevention Research Center Partnership Trust Tool (2019) [25], we asked participants to rate the importance (i.e., how important each attribute is to their organization) and quality (i.e., how well each attribute was applied within the partnership) of 12 components of trust (i.e., Values differences, Truthful, Supportive, Shares power/responsibilities, Responsible, Relationship building, Provides accurate information, Openness, Mutual Benefit, Good/Clear communication, Dependability, Accessibility) as experienced in their EHE partnership. For each component, participants used a 5-point Likert scale to rate each attribute’s perceived importance (1 = Not at all important, 2 = Little importance, 3 = Neutral, 4 = Important, 5 = Very important) and perceived quality (1 = Poor, 2 = Fair, 3 = Good, 4 = Very Good, 5 = Excellent). As intended in the original tool, each attribute was assessed as an individual question, thus we did not calculate a single sum score for ‘trust.’ One qualitative, free text response question at the end of the list of trust attributes asked, “Are there any other components of trust that you think should be included or addressed in this survey? If so, please describe in the box below:”.

##### Community partner organization engagement level:

One question, taken from Key et al.’s (2019) Continuum of Community Engagement Model [9], asked participants to report the extent to which the community partner organization was engaged in the EHE project. The question provided definitions and examples for each ‘level of engagement’ as defined by the original authors. Response options included: 1 = No Community Involvement; 2 = Community-informed; 3 = Community Consultation; 4 = Community Participation; 5 = Community Initiated; 6 = Community-based Participatory Research; and 7 = Community-driven.

##### Community partner engagement in research activities:

Adapting Khodyakov et. al.’s (2013) Community Engagement in Research Index [23], all participants, whether academic or community partners, reported the extent to which they perceived community partner engagement in the following 12 research activities: grant proposal writing, conducting background research, choosing research methods, developing sampling procedures, recruiting study participants, implementing the intervention, designing interview and/or survey questions, collecting primary data, analyzing collected data, interpreting study findings, writing reports and journal articles, and giving presentations at meetings and conferences. Response options for each item included: community partners did not participate in this activity, community partners consulted on this activity, and community partners were actively engaged in this activity. Both community and academic partner participants completed this question.

Qualtrics (Provo, UT) was used to administer the final survey. All responses were stored in a secure, password-protected database.

### Procedures

We recruited participants for this study from July-December 2023. In collaboration with the HIV Implementation Science Coordination Initiative (ISCI) and EHE Regional Consultation Hubs (https://hivimpsci.northwestern.edu/ehe-implementation-science-hubs/), we advertised the study in three bi-weekly EHE newsletters and asked Hub directors to assist with recruitment through meeting-based announcements and emails.

We invited all EHE awardee principal investigators (PIs) and academic co-investigators (Co-Is) from the 102 EHE projects awarded in 2021 or 2022 for whom contact information was available to participate in the study. Email invitations provided EHE awardees with a brief description of the study and a summary of the aims. The study team only had access to academic PI/Co-I contact information; thus only academic partners were contacted initially. Emails included eligibility criteria and a link to the informed consent form and online survey. PIs/Co-Is were given the option to forward the email to a colleague within their academic study team who they felt best met the eligibility criteria for the study (if someone other than themselves). We asked participants to provide contact information for their community partner(s). Upon receipt of community partner contact information, we emailed the community partner to invite them to participate. Recipients of the community partner email were also encouraged to forward it to a colleague within their organization who they felt best met the study’s eligibility criteria (if other than themselves). The email to community partners included the same information described above and the same link to the online consent form and survey. We asked that only one representative from the community partner organization and one representative from the academic partner organization complete a survey for a given project.

If a response was not received within two weeks, Hub directors followed up with their respective awardees via email, phone or through direct contact (e.g., an announcement made during a meeting). The study team also followed up via email and/or phone. If clarification was needed regarding community partner name, contact information, or EHE project title, researchers followed up with survey respondents via email and/or phone after their survey was received. EHE project titles were used to match community partner and academic partner responses for the same project. To reduce potential response bias, all responses were anonymous and respondents did not have knowledge as to whether their partner completed the survey.

### Analyses

We used descriptive statistics to describe participant demographics and quality and frequency of trust components. We applied skewness and kurtosis tests to assess normality of EP-specific scores and total quality and frequency scores. We calculated summary statistics for EP-specific scores and total frequency and quality scores (mean, median, standard deviation [SD], interquartile range [IQR], min, max). We used non-parametric (Mann-Whitney U rank sum) tests to assess differences in EP-specific scores between responses from community partners and academic partners in our sample. In response to items under the *community partner engagement in research activities* measure [23], some participants reported “Not Applicable” or “N/A” for dissemination-related activities, indicating that these participants may have completed the survey before their teams had reached the dissemination phase of their study. To account for any effect this may have on study outcomes, we conducted a sensitivity analysis for the dissemination-related EPs: “Involve all partners in the dissemination process” and “Disseminate findings and knowledge gained to all partners.” We recalculated Mann-Whitney U rank sum tests among a subset of the total respondents – excluding responses from participants who reported “Not Applicable” or “N/A” for dissemination-related activities.

Paired differences in perceived quality of trust among community partners and academic partners of the same project were assessed for concordance (i.e., the proportion of trust attributes for which community partners and academic partners reported the same quality scores) and mapped onto paired plots. To assess whether level of engagement was associated with quality of meaningful engagement principles and/or trust and whether meaningfulness of engagement was associated with trust, we used Spearman rank correlations. Missing data were excluded from analyses. All quantitative analyses were conducted using Stata SE 18 (StataCorp, College Station, Texas). Qualitative data from free-text response questions were coded thematically by researchers (RD and LB) to elicit overarching concepts related to meaningful engagement and trust in partnerships.

## Results

From July to December 2023, we received 82 completed individual surveys including 53 (65%) surveys from academic partners and 29 (35%) surveys from were community partners. The completed surveys represented data from 57 of the 102 possible EHE projects (56%) from 2021 to 2022. Of these, 22 (39%) projects included responses from one academic partner and at least one community partner of the same project. Eighteen of these projects had a submission from exactly one academic partner and one community partner of the same project, allowing for 1:1 within-project paired analyses. A detailed summary of survey response rates and characteristics among responders and non-responders was previously published [12].

Among respondents who reported demographic data (n = 80), most self-identified as white (59%), non-Hispanic (75%) female (58%), with a median age of 40 years [IQR 36–45] (Table 1). Among community partner participants (n = 29), 38% identified as white, compared to 71% among academic partners; nearly half (45%) of community partners identified as being of Hispanic ethnicity compared with 14% of academic partners. Gender distribution among community participants was approximately even (48% females and 45% males, respectively) with two participants (7%) identifying as transgender women. The subset of data comprising responses from one academic partner and at least one community partner was similar in demographic makeup to the overall sample (see Supplemental Material, S2).

### Engagement Principles

**Figure 1:**
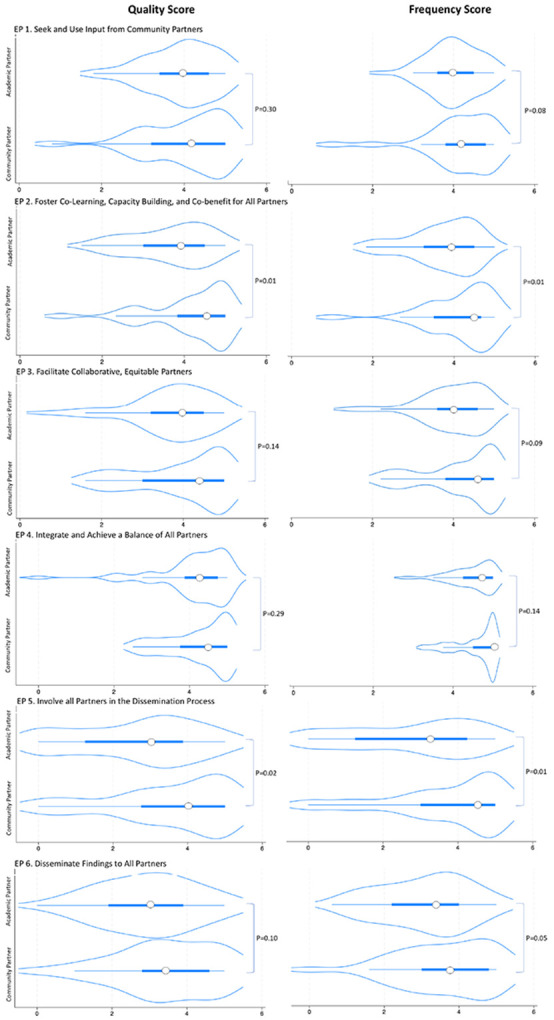
Violin plots display distribution of Engagement Principle (EP)-specific scores stratified by partner type (i.e., academic partner (n=52), community partner (n=29)). The white dot represents the median while the thick bar represents the interquartile range. The thin straight line represents the full distribution of responses. Distribution outlines denote kernel density estimation to show the distribution shape (i.e., wider sections represent higher probability that members of the partner type will select a given value; thinner sections represent a lower probability)

Overall, both academic and community partner respondents reported high frequency and quality scores for application of each of the EPs in their projects (see Supplemental Material, S3). Summary statistics of total quality and frequency scores indicate that academic partners reported lower median scores than community partners for both quality and frequency of EPs [3.6 (IQR = 3.0-4.1) vs 4.1 (IQR = 3.5–4.7), p = 0.04 and 3.8 (IQR = 3.3–4.2) vs 4.3 (IQR = 3.5–4.7), p = 0.02, respectively]. Among individual EPs, significant differences between academic and community partner assessment of engagement across all observations were reported for “Involving all Partners in the Dissemination Process” for both quality [−2.4, p = 0.02] and frequency [−2.5, p = 0.01] and for “Foster co-learning, capacity building, and co-benefit for all partners” for quality [−2.6, p = 0.01] and frequency [−2.6, p = 0.01]. While the differences were otherwise not significant, as shown in Fig. 1, academics generally reported lower quality and frequency scores than community partners for each EP.

### Sensitivity Analyses

Among the restricted subset of participants reporting having engaged in at least one dissemination activity (n = 54; 31 academic partners, 23 community partners), overall scores on relevant EPs were higher than in the full sample; however, academic partners consistently reported lower scores than community partners on dissemination-related Eps but here differences were not significant (see Supplemental Materials, S4). For the “Involve all partners in the dissemination process” EP, the analyses indicate differences in reported quality changed from − 2.4, p = 0.02 in the full dataset to −1.7, p = 0.09 in the restricted subset and differences in reported frequency changed from − 2.5, p = 0.01 in the full dataset to −1.8, p = 0.08 in the restricted subset. For the “Disseminate findings and knowledge gained to all partners” EP, the reported difference in quality changed from − 1.7, p = 0.10 in the full dataset to −0.9, p = 0.37 and the difference in frequency changed from − 2.0, p = 0.05 in the full dataset to −1.3, p = 0.21.

### Paired Analyses

Among the paired dataset (n = 36), no significant differences in quality or frequency were observed for any EPs. (see Supplemental Materials, S5). Academic partners perceived lower quality of application of all EPs than community partner respondents except for the frequency of “Facilitate collaborative, equitable partnerships” EP [0.35, p = 0.73].

### Open-text Responses

We received twenty open-text responses (15 from academic partners, five from community partners) in which respondents suggested measuring additional elements of meaningful engagement. From these responses, we identified several themes including: i) sociocultural responsiveness, ii) systematic barriers to meaningful engagement, iii) opportunities for interaction among partners, iv) community partner workload/burden, v) synergistic collaboration, vi) project initiation dynamics, vii) shared understanding of terms and structure and viii) receptivity of community partners to change and information. Structural barriers to meaningful engagement were a prominent theme. As one community partner noted,

Academic partners often design unrealistic studies, that somehow pass Center/NIH review, which then have to be implemented by community partners. Community partners are often burdened with fixing these studies and/or implementing a study they could have said from the outset would not be feasible. Due to rushed timelines for supplement submissions, there is little time for this kind of constructive criticism to be provided at the design phase, leaving community members feeling obligated to participate in a burdensome and difficult research project.

Another academic partner suggested including components of meaningful engagement related to how institutional structures facilitate or hinder partnerships:

Whether community partners and academic partners have the appropriate administrative support to facilitate grant funds, subcontracts is an important issue that should be assessed.

A full list of themes are summarized with exemplar quotes in Supplemental Material, S6.

### Partnership Trust

#### Quality of Partnership Trust

For each attribute of trust, ≥ 76% of academic partner respondents and ≥ 69% of community partner respondents reported “very good” or “excellent” partnership quality. Among academic partners, the trust attributes receiving the highest quality scores (i.e., highest proportion of “excellent” and “very good” scores) included valuing differences (n = 47, 90%), truthfulness (n = 45, 87%), supportiveness (n = 45, 87%), and accuracy (n = 45, 87%). Among community partners, attributes of valuing differences (n = 27, 93%), truthfulness (n = 26, 90%), and accessibility (n = 25, 86%) received the highest quality scores. Academic partners reported the lowest quality scores (i.e., attributes with the highest proportion of “poor” and “fair” scores) for dependability (n = 6, 12%), while community partners reported the lowest quality scores for mutual benefit (n = 3, 10%, see Supplemental Material, S7)

#### Importance of Partnership Trust

Overall, > 90% of academic partner respondents and > 86% of community partner respondents scored each attribute of trust as “important” or “very important.” Among academics, the attributes that received the highest proportion of “important” or “very important” scores included supportiveness and responsibility (n = 52, 100%, each). Among community partners, the attributes that received the highest proportion of “important” or “very important” scores included dependability, clear communication, accuracy, relationship building and responsibility (n = 28, 97% each). No attributes of trust were rated as having low importance (“Not at all important” or “Little importance”) by any participants, although “power sharing” received the highest proportion of “neutral” scores from both community partners (n = 3, 11%) and academic partners (n = 5, 10%, see Supplemental Material, S8)

#### Paired Comparisons

**Figure 2:**
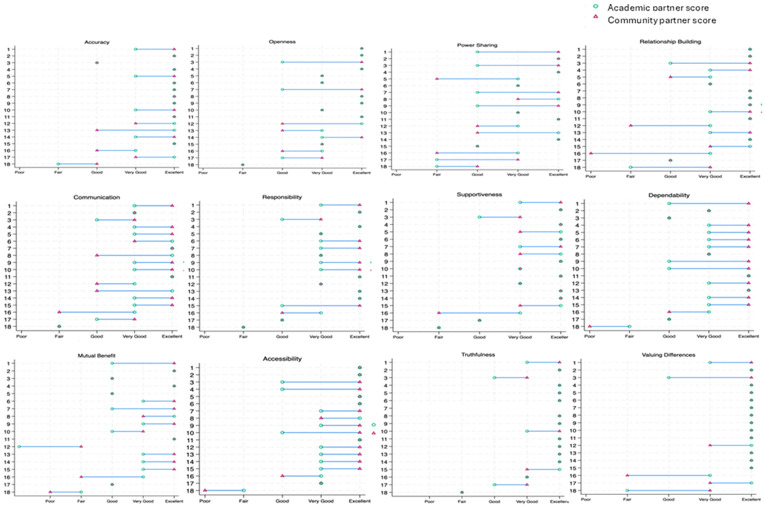
Differences in perceived quality of trust by project among paired partners (n=18 projects)

Among 18 pairs with responses from both a community and an academic partner working on the same project, we observed greatest concordance for quality of trust attributes for “truthfulness” (65% alignment) and “valuing differences” (67% alignment; Fig. 2) and least concordance for “Clear Communication” (22% alignment). All differences were two quality score points or fewer. There was little consistency in which partner type (community or academic) rated an attribute more highly (Fig. 2).

#### Open-text Responses

Three participants (two academic partners, one community partner) suggested measuring additional components of trust. From these, we identified two themes including: i) improving mutual understanding of research topics and methods, and ii) respect for partner’s time/boundaries. For example, one community partner suggested improved mutual understanding of research topics was an important trust attribute, stating, “When asked, [academic partner] explained complicated or new topics in a way that is easy to understand.” An academic partner described showing respect for partner’s boundaries saying, “Respect time of community partners (e.g., not overburdening community partners with tasks that research team could complete).” All identified components are summarized with exemplar quotes in the Supplemental Material, S6.

#### Level of community engagement and its association with quality of trust and engagement principles

Within trust attributes, Spearman’s rank correlation coefficients indicated reported level of community engagement was positively correlated with the quality of “Mutual Benefit” (.30, p = .03) among academic partners (Table 2). Among community partners, reported level of community engagement was positively correlated with quality of “Accessibility” (0.49, p = 0.01), “Dependability” (0.42, p = 0.03), “Openness” (0.53, p = 0.01), “Responsible” (0.41, p = 0.03) and “Shares Power” (0.46, p = 0.02). Among academic partners, level of community engagement was positively correlated with all six EPs (p < 0.05). Among community partners, level of community engagement was positively correlated with “Facilitate Collaborative and Equitable Partnerships” (0.39, p = 0.04), “Integrate and Achieve Balance of all partners” (0.40, p = 0.04), “Seek and use the input of community partners” (0.51, p = 0.01), and “Involve all partners in dissemination process” (0.48, p = 0.01).

#### Association between quality of engagement principles and quality of trust

Among academic partner responses, trust variables “Mutual Benefit,” “Relationship Building,” “Responsible,” and “Shares Power” were significantly positively associated (p < 0.05) with all six EP quality measures (Table 3). Among community partners, all 12 trust attributes were significantly positively correlated with all six EP quality measures (p < 0.05).

## Discussion

This study assessed perceived meaningful engagement and trust among community and academic partner recipients of EHE awards collaborating on HIV implementation science projects. Overall, participants reported largely meaningful and trusting partnerships. Community partners generally reported higher perceived quality and frequency of community engagement than academic partners across EPs, but scores were only significantly higher for the EPs of “involving all partners in the dissemination process” and “co-learning and co-benefit.” Among attributes of trust, academics and community partners reported their highest quality scores for “values differences” and “truthfulness” and their lowest for “dependability” and “mutual benefit”. Our results suggest that higher quality of key trust indicators are associated with a higher reported level of community engagement and higher perceived quality of engagement is correlated with higher quality of trust within the partnership.

Existing CEnR theoretical models and frameworks suggest that high quality engagement and trust in partnerships lead to more effective and equitable collaborative research processes [26-28]. For example, evaluations of CEnR projects using community-based participatory research (CBPR) models which aim to involve community members throughout all phases of research [29, 30]have highlighted the role of trust in achieving quality community-academic partnerships [14, 15]. Similarly, a study guided by the Model of Research-Community Partnership found trust among partners facilitated quality collaborative processes [18]]. Our findings are consistent with other studies that suggest trust is critical to effective, quality engagement [28, 31]. In the context of HIV IS, meaningful community engagement has the potential to aid in the achievement of health equity and facilitate the sustainability of successful programs and projects through development of strong and lasting collaborations [27]. Our results may inform strategies for improving community-academic partnerships via cultivating trust and advancing progress toward achieving EHE initiative goals.

Academic partners generally reported lower quality and frequency scores for the EP domains than community partners. Extant literature has reported on differences in community and academic partner perspectives regarding setting research priorities, expectations and goals [21, 22]. However, little research has evaluated differences in perceived quality of community engagement between community partners and academic partners. Acknowledging that academic partners have historically driven the design of research studies, one might expect community partners to report lower quality scores than academic partners. However, it is possible that given the wealth of research that has been conducted on CBPR in the academic space, academic partners may have been using a different (and perhaps more critical) set of criteria to determine the quality of engagement than community partners. Our previous work found that most community partners anticipated lower levels of community engagement prior to their partnership than they experienced [12]. If expectations started low and the experience was then perceived as a positive experience, it is possible that exceeding expectations may have boosted community partner’s scores. Additionally, social desirability bias among the community partners, who traditionally have less power, may have led to more favorable assessments. We observed the biggest differences between academic partners and community partners in responses related to “involving all partners in the dissemination process” and “fostering co-learning, capacity building and co-benefit for all partners.” While our survey alone does not explain the reasons for these disparities, the differences themselves highlight important priorities to discuss and address to support equitable partnerships. Community engagement literature demonstrates the importance of mutual benefit [32, 33]. The differences in perceptions highlight that early, open, and clear communication around what benefit different partners hope to derive from the collaboration should be a key priority. The traditional research system is structured such that standard methods of dissemination benefit academics directly, including publications and presentations that may lead to more funding, tenure, and/or promotion [34]. Recent literature has identified the importance of community-driven dissemination of research, particularly when the goal of implementation science is to increase application of evidence-based interventions [35, 36]. To ensure co-learning and benefit among partners, community engagement with research findings is critical [20, 37-39]. Further research to understand the etiologies of response differences is warranted. These differences indicate an opportunity to develop shared understanding of desired engagement quality and frequency, particularly in dissemination and co-learning practices.

While universally high, the trust attributes of “Dependability” and “Mutual benefit” received the lowest comparative quality scores for academic partners and community partners, respectively. Community partner organizations are often limited in their ability to contribute to research based on their own available resources, time, and technical capacity. As noted in other studies, there exists inherent structural differences between academic and community-based institutions including in organizational goals and staff turnover dynamics and frequency that may make it more difficult for community partners to reliably fulfill commitments [40, 41]. Further, community-based organizations partnering in research often must contend with competing priorities critical to their organizational mission, which can also serve as a barrier to consistent engagement with research. As noted through our qualitative insights, respect for community partners’ bandwidth and availability is critical in establishing trust and offers an acknowledgement of the innate challenges to dependability within the partnership. By respecting the time and capacity of community partners, researchers must offer flexibility to their community partners, making efforts to establish feasible timelines and commitments and ensure community partners are not overburdened or feeling pressured to work beyond their means. Moreover, it is critically important that researchers and community partners work together to identify what ‘benefits’ each partner wants from the research project. Our qualitative insights highlighted mutual understanding of research topics, methods, and findings as a key component of trust. Extant literature provides examples of strategies that have been used to build trust among community-academic partners including acknowledging unique expertise of all partners, developing shared goals or priorities, and demonstrating transparency around expected roles and responsibilities [42].

The more research activities community partners were engaged in, the higher the scores for perceived quality of trust and meaningful engagement. This is aligned with foundational studies of CBPR [29] and further validates the idea that increased engagement in all stages of the research process may lead to improved quality of engagement and trust. It also offers some evidence of criterion validity. Importantly, however, not all partnerships are the same, and discerning which activities community partners find feasible and desirable to engage in is critical. Perhaps of more importance is ensuring that community partners have the option to engage in any part of the research process that they want and that funding and institutional systems maximize opportunities for shared decision making and support across the research activity spectrum. Our study provides empirical evidence consistent with existing CEnR theoretical models and frameworks that draw on associations between quality engagement and trust in community-academic partnerships [42], suggesting that strategies to improve trust in a partnership may lead to improved quality engagement (and vice versa). Similarly, several studies describe the strategies for building authentic and meaningful community-academic partnerships, including establishing shared goals and purpose among partners, sharing use of resources, paying attention to community history and implementing effective conflict resolution techniques [10, 16, 18, 21, 37, 42]. To accelerate progress toward ending the HIV epidemic in, it is critical that researchers understand perceptions of trust and meaningful engagement among partners and leverage that knowledge to inform strategies to improve these dimensions of community-academic partnerships.

### Limitations

Our study has several limitations. Interpretation of terms, concepts, and definitions across survey questions among individual respondents may have differed. This was brought up directly in our free-response questions with one community participant noting that the term “community” in the survey question seemed to be different from their personal description of their own role. As noted in other studies, common terms and definitions used in CEnR are often interpreted or perceived differently among academic and community partners and even among individuals of the same partner organization type [17, 22, 23, 43]. Although we adapted a previously validated scale for measuring EPs [24] and limited trust attributes to simple, one-word terms, it is possible that differences in reported scores may be an artifact of differing perceptions of the questions themselves. Subsequent qualitative work will explore how these terms are interpreted across community and academic partners. To reduce respondent fatigue, we selected a sub-set of EPs from a larger validated scale that our research team felt were most pertinent to EHE projects. However, this may have limited the breadth of concepts related to meaningful engagement that our survey measured. Similarly, the attributes of trust outlined in the CDC’s Partnership Trust tool may also have gaps. To provide an opportunity for participants to voice additional components of meaningful engagement and trust that were important to them, we included an open text option in our survey. In comparing emergent topical themes with the original EP scale developed by Goodman at colleagues (2017) [24], there was some overlap. For example, participants in this study highlighted components related to “sociocultural responsiveness” which aligns with the “Acknowledge the community” EP in the original scale. Similarly, “synergistic collaboration” aligns with the “Build on strengths and resources within the community” EP in the original scale. However, not all qualitative findings aligned with original scale items. Some point to distinct themes related to institutional structures functioning as barriers/facilitators to meaningful engagement and respect for partners’ time and boundaries. The measures used in this study pertain to interactions and relationships between the academic team and the community partner project team but do not extend to relationships between individual partners nor between entire academic institutions (Universities) and the greater community setting. As noted by Christopher et al (2008), this is an important distinction [42]. At recruitment initiation, our study team only had access to academic partner contact information. Thus, our ability to recruit community partner participants in our sample was dependent on responsiveness from academic partners. This may have resulted in lower community partner participation in our study as linkage from academic partners to community partners was lower than anticipated. Finally, given the cross-sectional nature of this study, we were unable to evaluate the dynamic nature of partnerships. Longitudinal studies would allow researchers to explore how such partnerships change over time and how they can lead to sustained, mutually beneficial relationships.

### Implications

Taken together, these results suggest that EHE awards supported largely meaningful and trustworthy community-academic partnerships. To develop systematic and reliable measures for key CEnR concepts and processes, more research is needed to better understand how these terms may be interpreted by academic and community partners to develop generalizable, systematic and reliable measures for key CEnR concepts and processes. As acknowledged in other studies, there is an urgent need to address power imbalances between partners and ensure mutually beneficial partnerships to promote greater equity in the implementation science landscape [34, 40]. Future research should address these concepts. Correlations between quality of engagement principles and quality of trust attributes suggest that deliberate strategies to strengthen trust in partnerships may strengthen quality of community engagement and vice versa. While best practices for improved quality of partnerships have been described in prior studies [40, 44] and guides and recommendations have been provided for both community partners and academic partners [37, 44, 45], quantitative tests of such strategies on measures of quality of trust and engagement are lacking in the current evidence base. Understanding the impacts of these partnerships on longer-term outcomes such as sustainability and equity will only be possible if resources are available to sustain research partnerships and robust implementation infrastructure.

## Conclusions

This study contributes to the existing evidence base through its identification of key opportunities to improve community-academic partnerships to advance future HIV implementation science. The differences in perceived engagement among academic partners and community partners indicate an opportunity to develop greater shared understanding of desired engagement quality and frequency, particularly in dissemination and co-learning practices. Future efforts to improve partnerships and institutional systems will benefit from ongoing, in-depth reflection and evaluation of these long-term collaborations.

## Supplementary Material

This is a list of supplementary files associated with this preprint. Click to download.

• 08Jan2025CEnR2supplement.docx

## Figures and Tables

**Table 1:** Demographics among Community and Academic Participants

	Academic (n = 51)*		Community (n = 29)		Total (N = 80)	
Participant Characteristics	n	(%)	n	(%)	n	(%)
**Age (Median; [IQR])**	42	[37, 47]	39	[35, 42]	40	[36, 45]
**Race**						
**American Indian or Alaska Native**	0	(0.0)	1	(3.5)	1	(1.3)
**Asian**	6	(11.8)	1	(3.5)	7	(8.8)
**Black or African American**	5	(9.8)	5	(17.2)	10	(12.4)
**White**	36	(70.6)	11	(37.9)	47	(58.8)
**Native Hawaiian or Pacific Islander**	1	(2.0)	0	(0.0)	1	(1.3)
**Multiple Races**	3	(5.8)	9	(31.0)	12	(15.0)
**N/A**	0	(0.0)	2	(6.9)	2	(2.4)
**Ethnicity**						
**Hispanic**	7	(13.7)	13	(44.8)	20	(25.0)
**Non-Hispanic**	44	(86.3)	16	(55.2)	60	(75.0)
**Gender**						
**Female**	32	(62.7)	14	(48.3)	46	(57.5)
**Male**	18	(35.3)	13	(44.8)	31	(38.8)
**Non-binary**	1	(2.0)	0	(0.0)	1	(1.3)
**Transgender woman**	0	(0.0)	2	(6.9)	2	(2.4)
**Educational Attainment**						
**Less than high school diploma**	1	(2.0)	0	(0.0)	1	(1.3)
**High school diploma**	0	(0.0)	2	(6.9)	2	(2.4)
≥ **1 year(s) of college no degree**	0	(0.0)	7	(24.1)	7	(8.8)
**Bachelor's degree**	2	(3.9)	7	(24.1)	9	(11.3)
**Graduate degree**	48	(94.1)	13	(44.9)	61	(76.2)

* 2 missing

IQR: Interquartile range

N/A: ‘Not Applicable’

**Table 2:** Spearman's rank correlation coefficients

Trust Quality Measures	Engagement Level (Academic n = 52)	p- value	Engagement Level (Community n = 28)	p- value
Accessibility	0.19	0.18	0.49*	0.01
Dependability	0.18	0.19	0.42*	0.03
Clear Communication	0.03	0.82	0.36	0.06
Mutual Benefit	0.30*	0.03	0.38	0.05
Openness	0.14	0.33	0.53*	0.01
Accuracy	0.28	0.05	0.23	0.24
Relationship building	0.26	0.06	0.30	0.12
Responsible	0.20	0.16	0.41*	0.03
Shares power	0.25	0.07	0.46*	0.02
Supportive	0.19	0.17	0.37	0.06
Truthful	0.20	0.15	0.14	0.49
Values Differences	0.25	0.07	0.28	0.15
Engagement Principles (Quality)				
Facilitate Collaborative and Equitable Partnerships	0.38*	0.01	0.39*	0.04
Integrate and Achieve Balance of all partners	0.30*	0.03	0.40*	0.04
Seek and use the input of community partners	0.47**	0.00	0.51*	0.01
Foster co-learning, capacity building and co-benefit for all partners	0.32*	0.02	0.35	0.07
Involve all partners in dissemination process	0.28*	0.04	0.48*	0.01
Disseminate findings and knowledge gained to all partners	0.30*	0.03	0.37	0.05

* p < 0.05

** p < 0.01

*** p < 0.001

**Table 3.** Heat map display of association between quality of engagement principles and trust

	Quality of Engagement Principles											
	Academic Responses (n=52)						Community Responses (n=29)					
Trust Variables	Facilitate Collaborative and Equitable Partnerships	Integrate and Achieve Balance of all partners	Seek and use the input of community partners	Foster co-learning, capacity building and co-benefit for all partners	Involve all partners in dissemination process	Disseminate findings and knowledge gained to all partners	Facilitate Collaborative and Equitable Partnerships	Integrate and Achieve Balance of all partners	Seek and use the input of community partners	Foster co-learning, capacity building and co-benefit for all partners	Involve all partners in dissemination process	Disseminate findings and knowledge gained to all partners
Accessibility	0.22	0.60**	0.51**	0.35*	0.04	0.18	0.55*	0.59*	0.54**	0.52*	0.53*	0.38*
Dependability	0.38*	0.60**	0.53**	0.44*	0.19	0.21	0.42*	0.48*	0.54*	0.53*	0.46*	0.36
Clear Communication	0.31*	0.61**	0.47**	0.40*	0.13	0.25	0.65 **	0.79 **	0.76 **	0.80 **	0.61**	0.41*
Mutual Benefit	0.51**	0.61**	0.64**	0.58**	0.33*	0.45**	0.66 **	0.73 **	0.780 **	0.72 **	0.69 **	0.56*
Openness	0.27	0.52**	0.51**	0.28*	0.05	0.23	0.79 **	0.81 **	0.75 **	0.79 **	0.68 **	0.61 **
Accuracy	0.20	0.39*	0.38*	0.26	0.13	0.11	0.63 **	0.71 **	0.65 **	0.75 **	0.48*	0.41*
Relationship building	0.44*	0.58**	0.46**	0.52**	0.37*	0.36*	0.81 **	0.81 **	0.75 **	0.74 **	0.72 **	0.67 **
Responsible	0.46**	0.51**	0.53**	0.38*	0.30*	0.41*	0.69 **	0.74 **	0.79 **	0.71**	0.64 **	0.47*
Shares power	0.40*	0.54**	0.52**	0.54**	0.31*	0.45**	0.75 **	0.83 **	0.81 **	0.76 **	0.72 **	0.66 **
Supportive	0.52**	0.52**	0.44*	0.47**	0.26	0.36*	0.71 **	0.83 **	0.79 **	0.72 **	0.76 **	0.67 **
Truthful	0.34*	0.43*	0.46**	0.33*	0.23	0.26	0.57*	0.67 **	0.59 **	0.65 **	0.51*	0.43*
Values Differences	0.24	0.40*	0.48**	0.20	0.12	0.09	0.66 **	0.68 **	0.63 **	0.64 **	0.54*	0.42*

* p<0.05

** p<0.001

Green shades indicate stronger correlations (closer to 1) and red shades indicate weaker correlations (closer to 0).

## Data Availability

The datasets used and/or analyzed during the current study are available from the corresponding author on reasonable request.

## Abbreviations

• ART: Antiretroviral therapy
• PrEP: Pre–exposure prophylaxis
• US: United States
• EHE: Ending the HIV Epidemic
• NIH: National Institutes of Health
• IS: Implementation Science
• ISCI: Implementation Science Coordination Initiative
• CEnR: Community–engaged Research
• PI: Principal Investigator
• Co: I–Co–Investigator
• IQR: Interquartile range
• N/A: Not applicable
• CBPR: Community–based participatory research
